# Mr. Bowie on Bronchocele in Lower Canada

**Published:** 1830-07-01

**Authors:** 


### BRONCHOCELE IN LOWER CANADA.*

IT appears by a paper from Mr. Bowie in our Glasgow contemporary that bronchocele is very frequent in Lower Canada, although so level a country, that "one may travel hundreds of miles without meeting an eminence worthy the name of a hill." It would also appear that the French peasantry are peculiarly subject to the complaint, for in many districts there is scarcely a French family but has some of its members affected with goitre. Mr. Bowie has not, so far as we can see, formed any decisive opinion with regard to the etiology of bronchocele, indeed we should wonder if he had, but the following observations bear upon the subject.

"An argument in favour of the opinion that this disease is caused by the use of snow water, may be found in the fact, that in Canada the ground is covered with snow for about six months of the year; and in some places, where the disease appeared to me to be more than usually common, the inhabitants, during that period, had no other water than that procured from melted snow. But there are other districts, which are supplied with water, either from springs or clear streams, where the affection is by no means uncommon. It is only occasionally indeed that springs are resorted to by the peasants, by far the most common method of supplying themselves being from the neighbouring brook or river; and should there not be sufficient depth, to allow them to procure the requisite quantity from below the ice, they then have recourse to the gathering of snow, which is melted for domestic use.

"It is a fact, notorious amongst the residents in Canada, that the disease is mostly confined to the French peasants, and never appears amongst the natives of Britain, though they have been many years in that country. I have known it, however, amongst their children, although rarely. The difference in the manner of living, among the latter, and the greater attention to diet and other comforts, may help to explain how they are more exempt from the disease than the former. The French peasants are a hardy easy minded race, who care little about the kind of food or clothing they make use of; bacon, which they generally rear themselves, constitutes the principal article of their diet; if they have this, either made into bouil-

---

* Glasgow Med. Journ. No IX.

lon, or eaten with a piece of bread and a little rum, they are perfectly contented. This manner of living, no doubt, tends to produce a grossness of habit, of which there is no want of proof, to any person, who has had an opportunity of seeing, and treating their diseases.

" Again, the disparity of seasons in that climate, may have some influence in arousing affections, which, in a more uniform temperature, and better regulated habits, might for ever have lain dormant. In a climate, where in summer it is no unusual occurrence for Farenheit's thomemeter, placed in the shade, to rise as high as 96°, and in winter to sink to 25° and 30° below zero, one would suppose that a considerable modification of dress would be absolutely necessary. But this the Canadian in a great measure disregards. He may be seen in summer in his jacket and trowsers of coarse homespun, and his *bonnet rouge* on his head, and in winter he makes little addition, except a great coat of the same stuff, with a sash for the middle; but these are so carelessly put on, that they appear to be worn more from fashion, than from any necessity arising from the weather. I have frequently seen so much indifference displayed by these people, that in the coldest weather, they would drive over the snow, with their clothes so loosely fastened, that their bare bosoms would be exposed to a piercing atmosphere. That this exposure to the inclemency of the weather is calculated to call into action the predisposition to the disease may be inferred from the fact, that the higher classes of society, are comparatively little affected,—the English settlers never, and their children very rarely."

We fancy that few are now inclined to attribute very much in their theory of causation to the snow-water, but that the water in one way or other operates in the production of goitre, we have ever maintained and do still believe. Diet may have some effect in certain places, but in the Alpine regions, the cradle of the cretin and the land of goitre, nothing is more certain than that children fed in very various ways are almost equally liable to bronchocele. With regard to the exposure of the Canadian to atmospheric changes we shall simply observe that such exposure does not produce bronchocele elsewhere, nor is it probable that of itself it should prove a material cause of the disease. Mr. Bowie believes that it is connected with a scrofulous diathesis, and as there is no great novelty in this opinion we need dwell on it no longer. We pass to another point.

" With regard to the treatment of goitre, it is unnecessary to enlarge on the different remedies which have been employed for this purpose. It may be sufficient to state, that I have tried almost the whole of them, and never saw any good effects produced except from the iodine. So extraordinary have I found the efficacy of this remedy, that frequently while the tumour was rapidly disappearing under its influence, I have purposely stopped it, and substituted some such remedy as the potash, and most commonly the curative action was immediately suspended, and no farther progress made until the iodine was resumed.

" It seldom happens, that the Canadian peasants apply for medical advice at an early period; it is only after their own receipts have been long made use of in vain, and the complaint existed probably for a number of years, and attained such a size, as to become a deformity, that they consent to apply to the surgeon. Their backwardness will appear the more excusable, when we consider that until very lately, they were sceptical as to the power of medicine in the cure of bronchocele. This want of faith, however, is rapidly disappearing, and a belief, that medicine has some influence over this hitherto intractable complaint, is now manifested by those children of the forest, who form an opinion from the effects which they themselves have seen produced, without regard to traditionary prejudices.

" Very few cases that were not of longer standing than five or six years, failed either to be cured, or very materially alleviated, by a sufficiently long continuance in the use of iodine. The form used, was that of tincture, made by dissolving 48 grains in an ounce of spirit of wine. It was generally commenced in the dose of ten drops, and gradually increased until it reached thirty, three times a day. The time that elapsed before any effect was produced, was very various; sometimes there was a diminution in about eight or ten days; at others it was a month or two, before much progress was made. Occasionally a case occurred, over which the tincture of iodine seemed to have little control. In such, an ointment of the hydriodate of potash, rubbed on the swelling, was substituted, and often proved a powerful remedy. Very few cases occurred (with the exception of those of very long standing, where probably the derangement of the gland was so great, as to be altogether irremediable,) that withstood the use of these two remedies combined. In general, however, either of them was sufficient to effect a cure. It may be necessary to remark, however, that in recent cases, where pain and inflammation existed, the iodine or the ointment were not primarily employed. In such cases, it was found necessary to adopt an antiphlogistic plan of treatment, until the inflammation had abated. It was when the tumour had become somewhat indolent, that the beneficial effects of this specific were most marked."

When goitre is endemic and prevails to some extent it would appear to be more under the influence of iodine, than in counties or districts where it is sporadic only. At least, if this be not the fact, it will be difficult to account for the discrepancy of results between the iodine practice in Switzerland, and in Mr. Bowie's hands in Canada, and those obtained in this country. In the former it is hailed as almost a specific, in England it is a valuable remedy, but by no means infallible. We have witnessed its failure in several instances, and the statements in Dr. Bardsley's recent Hospital Reports, are very decisive on this point. This may be unpalatable to enthusiasts.

---

VIII.

